# Distribution and Potential Metabolic Functions of Soil Actinobacteria in Degraded Alpine Grassland on the Northern Tibetan Plateau

**DOI:** 10.3390/microorganisms13102230

**Published:** 2025-09-23

**Authors:** Junze Zhang, Sicen Luo, Yanying Wang, Yebing Yin, Yu Li, Wenxiang Zhao, Shirui Zheng, Guoqi Xu, Hongmei Ma, Pengxi Cao, Yixuan Liu

**Affiliations:** 1 Key Laboratory of Biodiversity and Environment on the Qinghai-Tibetan Plateau, Ministry of Education, School of Ecology and Environment, Xizang University, Lhasa 850000, China; zsemail@foxmail.com (J.Z.); sicen.luo@outlook.com (S.L.); yan.ying.wang@outlook.com (Y.W.); yvetteyy.yin@foxmail.com (Y.Y.); yuli8682@foxmail.com (Y.L.); zhaowenxiang09@outlook.com (W.Z.); shirui.zheng@outlook.com (S.Z.); xuguoqi1995@outlook.com (G.X.); hongmei.ma@foxmail.com (H.M.); pxcao@utibet.edu.cn (P.C.); 2 Lhasa Plateau Ecosystem Research Station, Key Laboratory of Ecosystem Network Observation and Modeling, Institute of Geographic Sciences and Natural Resources Research, Chinese Academy of Sciences, Beijing 100101, China; 3 Yani Wetland Ecosystem Positioning Observation and Research Station, Tibet University, Lhasa 850000, China

**Keywords:** alpine grassland, degraded, actinobacteria, northern Tibetan Plateau, 16S rRNA

## Abstract

Actinobacteria play major roles in human health and soil nutrient biogeochemical cycles, which are important for environmental protection. On the northern Qinghai–Tibet Plateau, the Qiangtang Alpine Grasslands have recently become degraded to varying degrees due to global climate changes and human disturbances. Here, we compared the community diversity, composition, and potential metabolic functions of Actinobacteria in soil from different degradation conditions through Illumina MiSeq sequencing. The soil Actinobacteria community structure in the Qiangtang Alpine Grasslands of northern Tibet was dominated by Nocardioides, Gaiella, Solirubrobacter, and Pseudonocardia, with evidence of previously unidentified taxa. Compared with non-degraded and severely degraded grasslands, the moderately degraded grassland had the highest soil Actinobacteria alpha-diversity, and the community composition showed significant differences between degraded grasslands with different degrees of degradation. The interactions between Actinobacteria and KO functions predominantly show negative correlations, but significant positive correlations outnumber significant negative ones. This study showed that Actinobacteria diversity and potential ecological functions in the alpine grasslands of northern Tibet decreased with grassland degradation due to the loss of vegetation cover. Therefore, it is necessary to effectively manage and protect the Qiangtang Alpine Grasslands on the northern Tibetan Plateau.

## 1. Introduction

Plant succession can significantly affect soil microbial community structure [1,2]. Soil microorganisms to a certain extent influence the cycling of carbon, nitrogen, and other substances [3,4], but they are dynamic and can rapidly adapt to the degradation of grasslands [5,6]. As a major group of soil microorganisms, Actinobacteria play important roles in regulating global chemical cycles, fixing mineral nutrients, improving soil fertility, and protecting the ecological environment [7]; they thus have important human health impacts. Actinobacteria produce many secondary metabolites and various bioactive substances. Indeed, approximately 40% of the known microbial bioactive substances and 70% of the antibiotics used in clinical practice are produced by Actinobacteria [8,9]. Furthermore, in a global study, Actinobacteria were found to be distributed in nearly half of all soil samples, with a relative abundance of more than 30% [10]. However, few studies have specifically investigated the ecological functions of Actinobacteria in soils [11]. Grassland degradation leads to the loss of biodiversity and the deterioration of ecosystem functions. Consequently, soil microbial community composition changes along with the soil quality index (SQI). The SQI value in degraded grasslands is lower than that of healthy meadow grasslands [12], indicating that grassland degradation significantly reduces soil quality and affects the diversity of soil microbial communities. The microbial activities and composition of grassland ecosystems are sensitive and quickly respond to changes in plant population structure and diversity [13]. Therefore, the impact of external pressures on grassland ecosystems is reflected by soil microbes. Studies comparing the community structure of actinomycetes in different ecosystems found that the relative abundance of actinomycetes in nutrient-poor deserts was higher than that in grasslands [14]. Yet, other studies have shown that Actinobacteria abundance is positively correlated with the carbon and nitrogen contents in soil [2,15,16].

The Qinghai–Tibet Plateau (QTP) is a global hotspot for biodiversity research [17]. High altitude, low oxygen content, strong ultraviolet radiation, and a large temperature difference between day and night endow the region with unique soil microbial resources that remain relatively understudied [2,18]. However, due to its high altitude, extreme temperatures, low rainfall, and relatively few vegetation species, QTP is particularly sensitive to global climate change. On the northern QTP, the Qiangtang Alpine Grasslands are composed of grasslands with three degradation levels: (1) non-degraded (ND), (2) moderately degraded (MD), and (3) severely degraded (SD); they have suffered disturbances of various degrees due to climate change and increased human activities [19,20]. Previous studies showed the grassland degradation alters plant community structure and plant diversity [21,22]. ND vegetation is rich in species and dense in growth. It is mainly composed of *Kobresia pygmaea*, *K. pusilla*, and *Poa arctica*. The commonly associated species of MD are *Stipa purpurea*, *S. subsessiliflora*, *Carex moorcroftii*, and *Androsace tibetica var. mairae (Kanitz) R. Knuth*. The vegetation coverage rate of SD is 15–20%, with a single-community composition mainly by *S. subsessiliflora*, *S. roborowskyi*, and *Oxytropis microphylla* [22,23].

This study not only provides foundational data for the comprehensive utilization of microbial resources in the Tibetan region but also offers a fundamental theoretical basis for the conservation of alpine meadows in the area.

## 2. Materials and Methods

### 2.1. Study Sites and Soil Sampling

**Figure 1:**
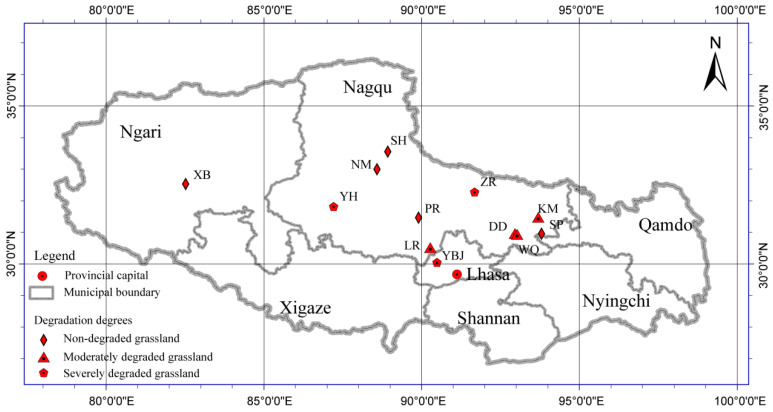
Sampling Sites. Thirty-six samples were collected from 12 sites in alpine grasslands of northern Tibet with different degrees of degradation.

In this study, soil samples were collected from 12 sites with different degrees of disturbance across the Qiangtang Alpine Grasslands of northern Tibet in August 2018. Three replicates per sample site were collected with a repetition interval of over 100 m; a total of 36 samples were collected (Figure 1). The 12 sites were divided into three ecosystem types with different degrees of degradation, namely ND, MD, and SD. Detailed information about the sample sites is provided in Table 1, and the criteria for distinguishing between three different degrees of grassland degradation are shown in Table 2 [24]. For each sample site, surface soils (0–10 cm) without plant cover were collected, homogenized, and stored in sterile bags. Samples were kept at −20 °C (Meigu Mobicool, Beijing, China, CF-50) during transport to the laboratory and then stored at −80 °C (Jiangsu Shenglan, Nanjing, China, DW86L-158) until DNA extraction.

### 2.2. DNA Extraction, PCR, and Illumina Sequencing

Total microbial DNA was extracted from soil samples using the E.Z.N.A.^®^ Soil DNA Kit (Omega Bio-Tek Inc., Norcross, GA, USA) according to the manufacturer’s recommended protocol. DNA quality and concentration were measured by a Nanodrop 2000 Spectrophotometer (Thermo Scientific, Waltham, MA, USA). The V3-V4 region of bacterial 16S rRNA genes was amplified with the primers 338F (5′-ACTCCTACGGGAGGCAGCAG-3′) and 806R (5′-GGACTACHVGGGTWTCTAAT-3′). Refer to previous studies to set PCR amplification conditions [25]. The amplicons were paired-end sequenced on the Illumina MiSeq platform according to the 300 bp protocol by Majorbio Bio-Pharm Technology Co., Ltd. (Shanghai, China).

### 2.3. Sequence Processing and Diversity Analysis

Raw reads were filtered by Trimmomatic software (version 0.33) to obtain high-quality clean reads. Reads with low average quality scores (<20) and short lengths (<100 nucleotides) were removed. Chimeras were checked and filtered by Fastp software (version 0.19.6). All downstream analyses performed after samples were rarefied to 19,234 sequences per sample. Then, effective tags were clustered into operational taxonomic units (OTUs) at similarities of 97% using Uparse version 7.1 (http://drive5.com/uparse/; accessed on 12 September 2021), and their taxonomies were assigned according to Silva’s database release 132 (http://www.arb-silva.de; accessed on 12 September 2021). Actinobacteria sequences were extracted by QIIME with the RDP Classifier (version 2.2).

### 2.4. Functional Prediction and Correlation Analysis

Phylogenetic Investigation of Communities by Reconstruction of Unobserved States was used to normalize OTUs, and the Kyoto Encyclopedia of Genes and Genomes (KEGG) database was used to predict potential metabolism functions of Actinobacteria. Networkx (version 2.5) was used to calculate the correlation between Actionbacteria community structure and metabolism functions and to construct the correlation network.

### 2.5. Statistical Analysis

QIIME was used to calculate the alpha-diversity indices (ACE, Chao1, Shannon, and Simpson) and beta-diversity distance matrix [26]. Analysis of similarities (ANOSIM) was used to compare whether the difference between groups was significantly greater than that within groups to assess significant groups. Hierarchical clustering analysis was conducted according to the Bray–Curtis dissimilarity, and the tree structure was constructed with the unweighted pair-group method with arithmetic mean algorithm. One-way analysis of variance (ANOVA) was performed to analyze differences in species diversity between groups. R software (version 2.15.3) was used to generate statistics and diagrams [27].

The results of this study have been submitted to the GenBank database under accession number PRJNA700796.

### 2.6. Determination of Soil Physical and Chemical Factors

To determine the pH value of soil using the potentiometric method, weigh 10 g of air-dried soil sample, add 25 mL of purified water, stir for 5 min, let stand for 1–3 h, and measure using a calibrated pH meter. To determine soil moisture content (SM) using the drying method, weigh 5 g of fresh soil, place it in a constant-temperature drying oven at 105 ± 2 °C for 48 h, and calculate using the formula SM (%) = (5-dry weight)/5 × 100%. Measure the electrical conductivity (EC) using a conductivity meter (HANNA, Rome, Italy, HI98304) with a water-to-soil ratio of 3:1. Determination of organic matter (OM) using the potassium dichromate volumetric method and dilution thermal method, determination of available potassium (AK) by ammonium acetate extraction and atomic absorption spectrophotometry, determination of available phosphorus (AP) by sodium bicarbonate extraction and spectrophotometry, determination of ammonium nitrogen (AN) using the Nessler reagent colorimetric method. Measure altitude using a handheld GPS (GARMIN, Olathe, KS, USA, eTrex309X). Use a lux meter (HANNA, HI97500) to measure the illumination intensity (II) on site, relative humidity (RH), dew-point temperature (DT), and atmospheric temperature (AT) on site. Use a carbon dioxide concentration meter (HANNA, HI4005-03) to measure the atmospheric carbon dioxide concentration (CO2 concentration, CC) on site. Use a soil temperature meter (TopCloud Agriculture, Hangzhou, China, TRS-II) to measure the soil temperature (Soil Temperature, ST) on site. The results of the soil physical and chemical properties and environmental factor tests are shown in Table 3.

## 3. Results

### 3.1. Alpha-Diversity of Actinobacteria

**Figure 2:**
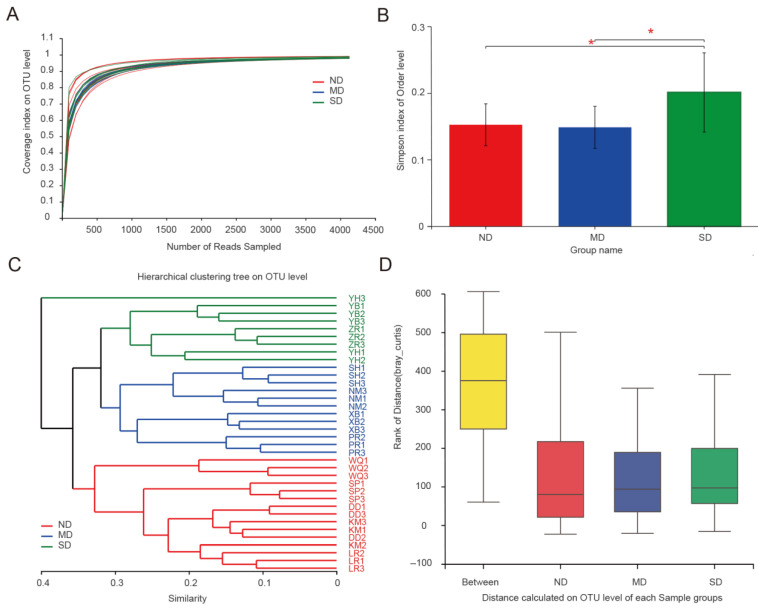
Alpha-diversity and beta-diversity analyses. (**A**) Rarefaction curve analysis. (**B**) Comparison of Actinobacteria alpha-diversity between degraded grasslands. Alpha-diversity was determined based on the Simpson index. (**C**) Hierarchical clustering analysis based on Bray–Curtis dissimilarity. (**D**) Analysis of similarities (ANOSIM) of intergroup and intra-group differences. MD, moderately degraded grassland; ND, non-degraded grassland; and SD, severely degraded grassland (* indicates a significant difference, *p* < 0.05).

A total of 1,713,518 effective sequences were obtained, from which 148,644 Actinobacteria sequences were extracted for OTU clustering and other follow-up analyses. In total, 799 Actinobacteria OTUs were obtained by clustering at 97% sequence similarity, with an average sequencing coverage of 98.19%. As the dilution curve tended to be flat (Figure 2A), the sequencing results effectively reflected the abundance of microbiota in the sample. Alpha-diversity indices of Actinobacteria in all samples are listed in Table 4. Boxplot analysis of the alpha-diversity indices of Actinobacteria among different ecosystems revealed that the Simpson index of the SD group was significantly higher than that of the ND and MD groups, while no significant difference was found between the Simpson indices of the ND and MD groups. (*p* < 0.01; Figure 2B). Hierarchical clustering showed that there are significant differences between samples originating from ND, MD, and SD. Except for SD’s YH3, all other samples were clustered into different branches according to their groups (Figure 2C). ANOSIM showed that there were differences between the groups, and the differences between groups were significantly greater than the differences within groups (*R* = 0.741, *p* < 0.001) (Figure 2D).

### 3.2. Community Composition of Actinobacteria

**Figure 3:**
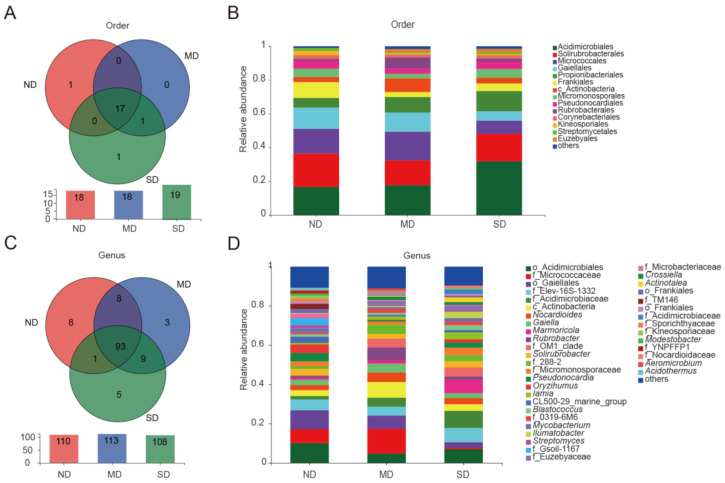
Composition and abundance analysis. (**A**) Venn map of three types of degraded grasslands at the order level. (**B**) Actinobacteria composition at the order level in each type of degraded grasslands. (**C**) Venn map of three types of degraded grasslands at the genus level. (**D**) Actinobacteria composition at the genus level in each type of degraded grasslands. MD, moderately degraded grassland; ND, non-degraded grassland; and SD, severely degraded grassland.

Based on the taxonomic assignments, Actinobacteria OTUs in the QTP soil samples were clustered into 20 orders, 63 families, 127 genera, and 254 species. In the three ecosystems, ND identified 18 orders and 110 genera of Actinobacteria, among them 1 order and 8 genera of Actinomycetes unique to ND; MD identified 18 orders and 113 genera, 3 genera of Actinomycetes unique to MD; SD identified 19 orders and 108 genera, 1 order and 5 genera of Actinomycetes unique to SD. The Actinobacteria found in common across the three ecosystems included 17 orders and 93 genera. Actinobacteria common to ND and MD include 8 genera, Actinobacteria common to MD and SD include 1 order and 9 genera, and Actinobacteria common to SD and ND include 1 genus (Figure 3A,C). At the order level, Acidimicrobiales was the most abundant population (average relative abundance: 22.04%), followed by Solirubrobacterales (16.95%), Micrococcales (13.12%), Gaiellales (9.84%), Propionibacteriales (8.97%), Frankiales (5.65%), Micromonosporales (4.31%), Pseudonocardiales (4.03%), and Rubrobacterales (3.14%). However, the relative abundance of these major taxa varied greatly among the different ecosystems. For example, the most abundant relative abundance in ND is Solirubrobacterales (19.78%), followed by Acidimicrobiales (17.41%), Micrococcales (12.54%), and Gaiellales (10.33%). In MD, the most abundant relative abundance is Acidimicrobiales (18.32%), the second is Micrococcales (16.98%), followed by Solirubrobacterales (15.31%), and Gaiellales (12.91%). In SD, the relative abundance of Acidimicrobiales was significantly greater than that of the other two groups, accounting for 32.47%, followed by Solirubrobacterales (16.67%), Propionibacteriales (7.63%), and Micrococcales (4.84%) (Figure 3B).

**Figure 4:**
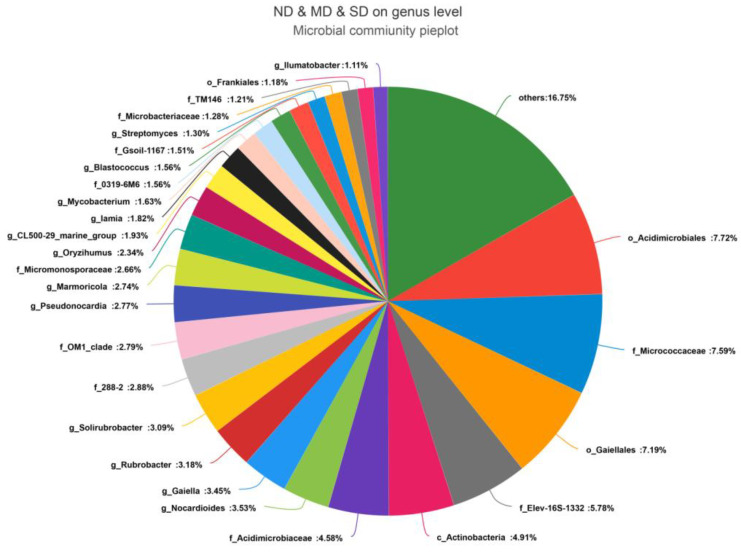
Actinobacteria community at the genus level with average relative abundance over 1%.

**Figure 5:**
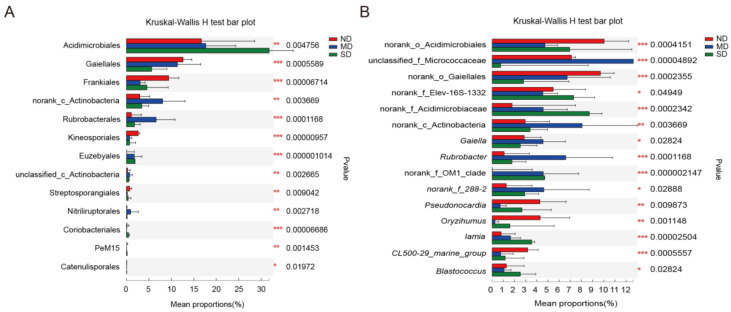
Analysis of differences among degraded grasslands based on the Kruskal–Wallis test. (**A**) Significance test of top 17 orders. (**B**) Significance test of top 27 genera. MD, moderately degraded grassland; ND, non-degraded grassland; and SD, severely degraded grassland. (* indicates a significant difference, *p* < 0.05; ** indicates a highly significant difference, *p* < 0.01; *** indicates extremely significant difference, *p* < 0.001).

At the genus level, unclassified strains accounted for 16.26% of the total number of OTUs. Apart from unclassified strains, 32 Actinobacteria genera with relative abundances exceeding 1% were identified in the ND group, dominated by Order Acidimicrobiales, Genus Unidentified (11.31%), Order Gaiellales, Genus Unidentified (9.05%), Family Micrococcaceae, Genus Unidentified (6.76%), and Family Elev-16S-1332, Genus Unidentified (5.31%). There are 25 Actinobacteria genera with relative abundances exceeding 1% in the MD group, primarily consisting of Family Micrococcaceae, Genus Unidentified (17.27%), Class Actinobacteria, Genus Unidentified (8.21%), *Rubrobacter* (6.51%), and Order Gaiellales, Genus Unidentified (5.37%). In the SD group, there are 30 Actinobacteria genera with relative abundances exceeding 1%, mainly including Family Acidimicrobiaceae, Genus Unidentified (9.36%), Family Elev-16S-1332, Genus Unidentified (8.47%), Order Acidimicrobiales, Genus Unidentified (7.72%), and *Marmoricola* (7.54%) (Figure 3D). Of the 93 genera identified in common across the three ecosystems, 27 had an average relative abundance of more than 1%, accounting for 80.77% of the total Actinobacteria, including *Nocardioides* (3.42%), *Gaiella* (3.34%), *Rubrobacter* (3.08%), *Solirubrobacter* (3.00%), *Pseudonocardia* (2.69%), *Marmoricola* (2.65%), *Oryzihumus* (2.27%), and *Iamia* (1.76%). Genera belonging to the top five most abundant orders accounted for similar percentages of the total number of OTUs, including *Acidimicrobiales* (20.78%), *Solirubrobacterales* (17.23%), *Micrococcales* (13.69%), *Gaiellales* (10.43%), and *Propionibacteriales* (8.45%) (Figure 4). Among the 17 orders and 27 genera commonly found with average relative abundances >1%, 13 orders—dominated by Acidimicrobiales, Gaiellales, Frankiales, Class Actinobacteria Order Unidentified, Rubrobacterales, and Kineosporiales—and 23 genera—dominated by Order Acidimicrobiales Genus Unidentified, Family Micrococcaceae Genus Unidentified, Order Gaiellales Genus Unidentified, Family Elev-16S-1332 Genus Unidentified, Family Acidimicrobiaceae Genus Unidentified, and Class Actinobacteria Genus Unidentified—showed significant differences between the three ecosystems (*p* < 0.05) (Figure 5A,B).

### 3.3. Predicting the Potential Function of Actinobacteria Communities

**Figure 6:**
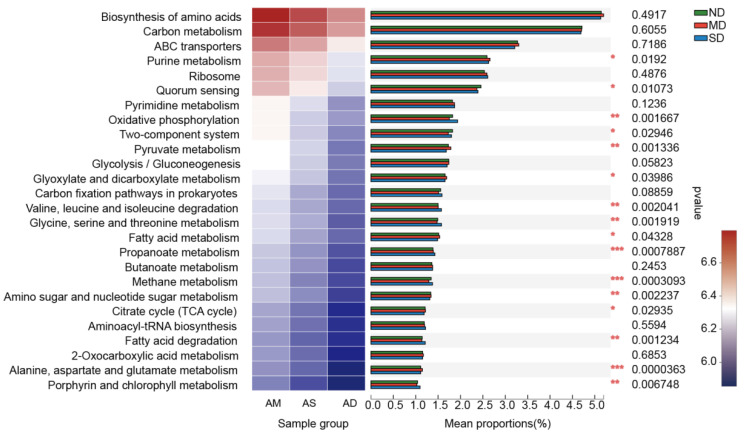
Significant difference analysis for metabolic functional classification of different genera in Actinobacteria phylum among degraded grasslands. MD, moderately degraded grassland; ND, non-degraded grassland; and SD, severely degraded grassland. (* indicates a significant difference, *p* < 0.05; ** indicates a highly significant difference, *p* < 0.01; *** indicates extremely significant difference, *p* < 0.001).

The nearest sequenced taxon index scores of all samples varied from 0.02 to 0.98, suggesting that the predictions were accurate and reliable. Among all potential functions predicted by the KEGG database, 22 KEGG Ortholog (KO) functions had a relative abundance of more than 1%, belonging to global and overview maps (12.53%); carbohydrate metabolism (11.51%); nucleotide metabolism (4.47%); amino acid metabolism (4.16%); energy metabolism (3.74%); lipid metabolism (1.16%); and cofactors and vitamins metabolism (1.05%). The global and overview map functions included amino acid biosynthesis (5.15%), carbon metabolism (4.71%), fatty acid metabolism (1.51%), and 2-oxocarboxylic acid metabolism (1.16%). The carbohydrate metabolism functions included pyruvate metabolism (1.74%); glycolysis/gluconeogenesis (1.73%); glyoxylate and dicarboxylate metabolism (1.67%); propanoate metabolism (1.4%); butanoate metabolism (1.37%); amino sugar and nucleotide sugar metabolism (1.33%); citrate cycling (1.21%); and starch and sucrose metabolism (1.06%). The nucleotide metabolism functions included purine metabolism (2.62%) and pyrimidine metabolism (1.85%). The amino acid biosynthesis functions included valine, leucine, and isoleucine degradation (1.52%); glycine, serine, and threonine metabolism (1.52%); and alanine, aspartate, and glutamate metabolism (1.12%). The energy metabolism functions included prokaryote carbon fixation pathways (1.55%), methane metabolism (1.34%), and oxidative phosphorylation (1.82%). The lipid metabolism function was only composed of fatty acid degradation (1.16%). The cofactors and vitamins metabolism functions were porphyrin and chlorophyll metabolism (1.05%). The ANOVA results also revealed that 14 of the above 22 metabolic functions showed significant differences between the three ecosystems (*p* < 0.05), namely, Purine metabolism; Quorum sensing; Oxidative phosphorylation; Two-component system; Pyruvate metabolism; Glyoxylate and dicarboxylate metabolism; Valine, leucine and isoleucine degradation; Fatty acid metabolism; Propanoate metabolism; Methane metabolism; Amino sugar and nucleotide sugar metabolism; Citrate cycle (TCA cycle), Fatty acid degradation; Alanine, aspartate and glutamate metabolism; and Porphyrin and chlorophyll metabolism (Figure 6).

### 3.4. Correlation Analysis Between Metabolism Functions and Actinobacteria Community

**Figure 7:**
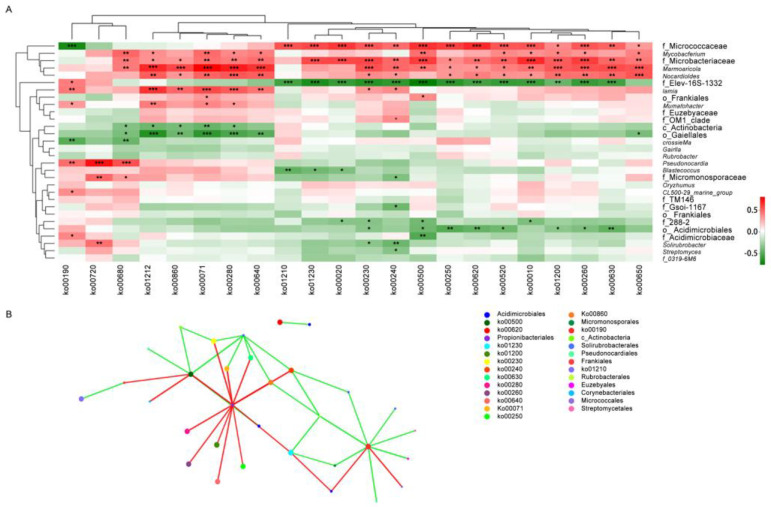
Correlation analysis between metabolism function and Actinobacteria community. (**A**) Correlation between metabolism functions and Actinobacteria at the genus level. (**B**) Interaction between metabolism functions and Actinobacteria at the genus level. ko01230, biosynthesis of amino acids; ko01200, carbon metabolism; ko00230, purine metabolism; ko00240, pyrimidine metabolism; ko00190, oxidative phosphorylation; ko00620, pyruvate metabolism; ko00010, glycolysis/gluconeogenesis; ko00630, glyoxylate and dicarboxylate metabolism; ko00720, carbon fixation pathways in prokaryotes; ko00280, valine, leucine, and isoleucine degradation; ko00260, glycine, serine, and threonine metabolism; ko01212, fatty acid metabolism; ko00640, propanoate metabolism; ko00650, butanoate metabolism; ko00680, methane metabolism; ko00520, amino sugar and nucleotide sugar metabolism; ko00020, citrate cycle (TCA cycle); ko00071, fatty acid degradation; ko01210, 2-oxocarboxylic acid metabolism; ko00250, alanine, aspartate, and glutamate metabolism; ko00500, starch and sucrose metabolism; and ko00860, porphyrin and chlorophyll metabolism. (* indicates a significant difference, *p* < 0.05; ** indicates a highly significant difference, *p* < 0.01; *** indicates extremely significant difference, *p* < 0.001).

Based on the correlation analysis between the top 27 genera and top 22 KO functions (relative abundance >1%), *Nocardioides* positively correlated with all KO functions. In addition to this, Family Micrococcaceae Genus Unidentified, *Mycobacterium*, Family Microbacteriaceae Genus Unidentified, and *Marmoricola* show positive correlation with nearly all KO functions. Notably, the significant positive correlation with KO function is also highly enriched across these 5 genera; additionally, significant positive correlations were also observed within *Iamia*, Order Frankiales Genus Unidentified, *Ilumatobacter*, Family OM1_clade Genus Unidentified, *Pseudonocardia*, Family Micromonosporaceae Genus Unidentified, *CL500-29_manine_group*, Family Acidimicrobiaceae Genus Unidentified, and *Solirubrobacter*. Class Actinobacteria Genus Unidentified, Order Gaiellales Genus Unidentified, Family 288-2 Genus Unidentified, Order Acidimicrobiales Genus Unidentified, Family Acidimicrobiaceae Genus Unidentified, *Solirubrobacter*, and Family 0319-6M6 Genus Unidentified exhibit negative correlation with the vast majority of KO functions. Significant negative correlations are markedly fewer than significant positive correlations, with significant negative correlations primarily enriched in Family Elev-16s-1332, Class Actinobacteria Genus Unidentified, Order Gaiellales Genus Unidentified, *Crossiella*, *Blastococcus*, Family Micromonosporaceae Genus Unidentified, Family Gsoil-1167 Genus Unidentified, Family 288-2 Genus Unidentified, Order Acidimicrobiales Genus Unidentified, *Solirubrobacter*, and *Streptomyces* (*p* < 0.01; Figure 7A). According to interaction between metabolism functions and Actinobacteria at the genus level result, those of *Pseudonocardia*, *Blastococcus*, *Oryzihumus*, *Solirubrobacter*, *Streptomyces*, *Solirubrobacterales*, and other no-rank genera in Acidimicrobiales positively correlated with energy metabolism but negatively correlated with other metabolic functions (*p* < 0.01). Those of *Crossiella*, *Gaiella*, and *Rubrobacter*, among others, negatively correlated with energy, fatty acid, propanoate, porphyrin, and chlorophyll metabolism. Overall, the negative correlation between Actinobacteria and KO function was more prevalent than the positive correlation, but the significance of the positive correlation was greater than that of the negative correlation. (*p* < 0.01; Figure 7B).

## 4. Discussion

*Acidimicrobiales* were found to be the most abundant Actinobacteria in the grassland soils of northern Tibet. Other studies reported that in the soils formed by retreating glaciers in Western China, *Acidimicrobiales* accounted for 19% of all bacterial groups [25], whereas the content of *Acidimicrobiales* increased significantly in the degraded grassland soils of northwest Yunnan [28]. Consistent with these previous reports, our study found that *Acidimicrobiales* increased gradually with the degradation of grasslands, with the highest abundances found in SD. *Solirubrobacterales*, a group of psychrotolerant Actinobacteria, were found to be the second most abundant Actinobacteria in the grasslands of northern Tibet, but only three genera containing 11 species have been previously described [29]. In our study, the abundance of *Solirubrobacterales* in ND was much higher than that in SD, indicating that additional related but so far uncultured taxa exist in nature and therefore requiring further research.

We also found that the abundance of *Gaiellales* decreased gradually as grassland degradation increased on the northern Tibetan Plateau. These organisms are known to assimilate sugars [30], myo-inositol, organic acids, and amino acids [31]. However, since large numbers of organic nutrients are not utilized by *Gaiellales*, their metabolic functions appeared relatively weak in our analysis. In contrast to that of *Gaiellales*, *Propionibacteria* abundance increased with grassland degradation. Moreover, the abundances of *Micromonosporales* and *Pseudonocardiales* were similar in both ND and SD but higher than those found in MD. However, the abundances of *Micrococcales*, *Rubrobacterales*, and a class of no-rank order of Actinobacteria were higher in MD than those in ND and SD. Research indicates that soil moisture content, organic carbon, total nitrogen, and total phosphorus levels decrease as grassland degradation intensifies [31]. These actinomycetes participate in the soil carbon and nitrogen cycles and metabolism, affecting nutrient cycling and energy metabolism under environmental stress [32]. Soil moisture content also significantly affects the abundance and diversity of soil microorganisms [33]. These factors led to differences in Actinobacteria abundance across the three degraded grasslands.

Actinobacteria have several important ecological functions, including decomposition of various organic substances, fixation of nitrogen, as well as improvement of soil physical and chemical parameters [34]. They also play an important role in plant diseases and their control [35]. Under environmental selection pressures, Actinobacteria, which grow and propagate in the extreme QTP environment, regulate their metabolic processes, form special physiological mechanisms, and produce secondary metabolites and various bioactive substances. Of the three degraded types of grassland in our study, we observed that the Actinobacterial metabolic functions with average relative abundances over 1% were the greatest in ND and the least in SD. We also reported that the metabolism of methane (KO00680) and propanoate (KO00640), as well as alanine, aspartate, and glutamate (KO00250) differed significantly between the three degraded types of grassland across northern Tibet. This indicates that the soil microbial contribution to carbon flux also varied.

In soils, microorganismal growth and survival are affected by various factors, for example, soil organic carbon, total nitrogen, and soil moisture content [33,36], which determine their utilization of different resources and their response to different environmental conditions. Recently, several microbial ecologists applied Grime’s Universal Adaptive Strategy Theory, namely the Competitors-Stress Tolerators-Ruderals (C-S-R) Triangle Theory of life-history strategies [37], to a microbial system and found that the system can predict species distribution to a certain extent [38,39,40]. In our study of the grassland soils on the northern Tibetan Plateau, we found that *Pseudonocardia*, *Blastococcus*, *Oryzihumus*, *Streptomyces*, CL500-29 marine group, and an unclassified genus in Frankiales with a high abundance of energy metabolism functional genes showed high abundances in ND. Meanwhile, the relative abundances of *Marmoricola*, *Mycobacterium*, *Nocardioides*, *Microbacterium*, and an unclassified genus of Micrococcaceae with high gene abundance in all 22 metabolic functions presented no relationship with the degree of grassland degradation. However, as the metabolism of microorganisms is diverse, and their survival in extreme conditions is extremely susceptible to environmental stresses and outside interference [41]. Additional experiments are necessary to verify the metabolic functions of soil microorganisms in the degradation process of alpine grasslands. In this study, we found a high diversity of Actinobacteria in the soils from three types of ecosystems in the Qiangtang Alpine Grasslands of northern Tibet. The community structure was dominated by *Nocardioides*, *Gaiella*, *Solirubrobacter*, and *Pseudonocardia*. However, with grassland degradation, the abundance and diversity of the Actinobacteria community changed significantly, and the abundance of genes related to their metabolic functions decreased.

This study also identified numerous unannotated fungal species, suggesting that northern Tibet’s degraded grasslands harbor substantial undiscovered microbial resources. Whether these fungal species perform critical ecological functions within degraded grasslands remains to be confirmed through further research.

## 5. Conclusions

Our results revealed that the Actinobacteria communities in the alpine grassland soils on the northern Tibetan Plateau are characterized by high taxonomic richness and diversity. We also found that Actinobacteria diversity declined as the ecosystem’s degree of degradation increased, with significantly higher values in ND and MD than in SD. In the soils of ND and MD, the relatively large volume of vegetative litter provides abundant organic matter, which is conducive to the growth and reproduction of Actinobacteria and promotes material cycling and energy flow. However, due to the loss of vegetation cover in SD and high variation in surface temperatures resulting in water and heat loss, Actinobacteria growth and reproduction was lower. This results in a significant positive correlation between Actinobacteria and their metabolic functions, enabling them to resist and compensate for negative impacts such as nutrient depletion, increased heat loss, and water loss in their habitats. Overall, Actinobacteria diversity and potential ecological functions in the alpine grasslands of northern Tibet decreased with grassland degradation due to the loss of vegetation cover.

## Figures and Tables

**Table 1:** Geographical data of collected samples in this study.

Degradation Degrees	Symbol	Location	Latitude	Longitude	Altitude (m)
Non-degraded grassland (ND)	KM	Kongma Township, Nagqu	31°28′50.85″	93°41′31.28″	4472
	LR	Longren Township, Nagqu	30°31′23.44″	90°16′19.71″	4292
	DD	Dongde Lake, Jiali County, Nagqu	30°57′59.58″	92°57′04.80″	4893
	WQ	Wuqiong Lake, Jiali County, Nagqu	30°57′59.54″	92°57′04.84″	4691
Moderately degraded grassland (MD)	PR	Puruogangri Glacier, Shuanghu County, Nagqu	31°28′18.33″	89°53′55.7″	5262
	SP	Sapu Glacier, Biru County, Nagqu	30°57′26.67″	93°48′01.64″	4722
	SH	Shuanghu County, Nagqu	33°33′33.61″	88°55′34.61″	4815
	XB	Xiongba Township, Bange County, Ngari	32°32′07.74″	82°31′13.42″	4475
	NM	Nima County, Nagqu	32°59′58.62″	88°35′15.29″	4784
Severely degraded grassland (SD)	YH	Yanhu Township, Geji County, Nagqu	31°49′15.54″	87°12′51.07″	4201
	YB	Yangbajing Town, Dangxion County, Lhasa	30°03′14.51″	90°29′19.90″	4173
	ZR	Zaren Township, Anduo County, Nagqu	32°16′52.22″	91°40′54.96″	4566

**Table 2:** Criteria for distinguishing between three different degrees of grassland degradation.

Degree of Degeneration	Plants Cover (%)	Proportion of Primary Plant (%)	Height of Plant (cm)
ND	80–95	70	25
MD	50–70	30–50	5
SD	<30	<15	-

**Table 3:** The physicochemical data of the samples collected in this study.

Ecotype	Sampling Site	pH	Soil Moisture (%)	Electrical Conductivity (ms/cm)	Organic Matter (mg/kg)	Available K (mg/kg)	Available P (mg/kg)	Available N (mg/kg)	Illumination Intensity (lx)	Relative Humidity (%)	CO_2_ Concentration (ppm)	Dew-Point Temperature (°C)	Atmospheric Temperature (°C)	Soil Temperature (°C)
Non-degraded grassland (ND)	KM	7.74 ± 0.15	0.47 ± 0.03	0.75 ± 0.13	214.03 ± 18.94	352.7 ± 11.65	34.82 ± 10.2	740.5 ± 41.36	56,656	50.6	928	8.3	17.8	13.6
	LR	5.64 ± 0.1	0.14 ± 0.01	0.01 ± 0.01	238.17 ± 19.89	73.25 ± 6.69	5.03 ± 4.11	915.03 ± 33.96	141,344	28.9	817	12.3	31.7	23.7
	DD	5.06 ± 0.02	0.29 ± 0	0.01 ± 0.01	170.23 ± 21.11	160.43 ± 29.51	22.65 ± 4.6	853.4 ± 32.19	133,650	25.4	827	5.8	27.4	12.9
	WQ	4.24 ± 0.05	0.31 ± 0.02	0.02 ± 0.01	160.67 ± 21.97	273.03 ± 36.98	12.04 ± 0.26	894.8 ± 54.77	155,000	43.8	783	6.1	17.9	11.4
	PR	8.18 ± 0.13	0.12 ± 0.01	0.08 ± 0.02	150 ± 17.06	8.22 ± 3.63	2.44 ± 1.39	590.07 ± 44.51	42,976	64.9	846	5.3	12	13.9
Moderately degraded grassland (MD)	SP	5.81 ± 0.04	0.22 ± 0.03	0.01 ± 0	200.97 ± 35.08	135.77 ± 31.12	7.07 ± 2.62	644.43 ± 59.05	43,600	41.6	799	4.4	16.9	16
	SH	8.12 ± 0.04	0.12 ± 0	0.05 ± 0.01	211.3 ± 14.25	148.3 ± 38.9	6.2 ± 1.85	768.63 ± 76.08	59,296	66.4	932	6.6	12.8	10.1
	XB	9.4 ± 0.03	0.1 ± 0.01	0.27 ± 0.02	68.28 ± 9.13	207.5 ± 22.51	7.1 ± 4.06	477.3 ± 29.88	31,644	30.8	712	5.2	22.6	18.8
	NM	8.56 ± 0.02	0.14 ± 0.01	0.22 ± 0	165.33 ± 16.97	196.8 ± 40.74	6.14 ± 0.88	672.77 ± 64.07	40,208	37.7	880	13.4	28.5	18.4
Severely degraded grassland (SD)	YH	7.21 ± 0.06	0.24 ± 0.03	0.11 ± 0.01	147.37 ± 10.6	211.37 ± 9.86	17.48 ± 2.29	804.4 ± 67.59	153,280	36.3	860	11.7	27.3	14.6
	YB	6.27 ± 0.14	0.14 ± 0	0.03 ± 0.01	125.23 ± 15.51	138.8 ± 28.14	12.22 ± 0.1	859.87 ± 45.61	60,896	31.3	806	9.1	26.3	22.8
	ZR	7.29 ± 0.02	0.47 ± 0.04	0.18 ± 0.03	277.03 ± 57.85	539.6 ± 7.31	35.28 ± 6.81	923.5 ± 79.1	28,037	62.9	850	13.4	13.5	11.1

**Table 4:** Alpha-diversity index table.

Sample\Estimators	Shannon	Simpson	ACE	Chao1
DD1	4.76 ± 0.01	0.02	397.38 ± 0.03	396.29 ± 0.04
DD2	4.84 ± 0.01	0.02	410.15 ± 0.02	397.66 ± 0.02
DD3	4.63 ± 0.02	0.03	396.08 ± 0.05	388.88 ± 0.04
KM1	4.90 ± 0.01	0.02	409.75 ± 0.01	421.34 ± 0.03
KM2	4.94 ± 0.01	0.01	405.51 ± 0.05	401.75 ± 0.01
KM3	4.53 ± 0.03	0.02	373.09 ± 0.02	379.08 ± 0.01
LR1	5.20 ± 0.01	0.01	430.85 ± 0.03	453.08 ± 0.04
LR2	4.75 ± 0.01	0.02	426.90 ± 0.03	436.92 ± 0.03
LR3	5.12 ± 0.02	0.01	475.39 ± 0.04	484.47 ± 0.01
SP1	4.48 ± 0.01	0.03	342.91 ± 0.02	333.78 ± 0.05
SP2	3.90 ± 0.01	0.08	337.85 ± 0.02	348.16 ± 0.03
SP3	4.20 ± 0.02	0.06	334.37 ± 0.01	328.00 ± 0.01
WQ1	3.94 ± 0.01	0.03	202.57 ± 0.05	203.00 ± 0.04
WQ2	4.04 ± 0.03	0.03	225.79 ± 0.04	229.00 ± 0.03
WQ3	3.97 ± 0.01	0.03	208.88 ± 0.03	209.22 ± 0.02
NM1	4.73 ± 0.01	0.02	389.70 ± 0.02	392.19 ± 0.02
NM2	4.84 ± 0.01	0.01	413.57 ± 0.01	436.41 ± 0.04
NM3	4.58 ± 0.03	0.02	369.20 ± 0.04	386.29 ± 0.04
PR1	4.46 ± 0.01	0.05	396.70 ± 0.01	389.02 ± 0.05
PR2	4.83 ± 0.01	0.02	421.27 ± 0.01	425.02 ± 0.01
PR3	4.71 ± 0.03	0.03	407.24 ± 0.03	406.42 ± 0.03
SH1	4.87 ± 0.01	0.02	404.72 ± 0.01	404.23 ± 0.02
SH2	4.66 ± 0.04	0.02	387.82 ± 0.02	388.00 ± 0.01
SH3	4.78 ± 0.01	0.02	423.81 ± 0.04	429.29 ± 0.03
XB1	4.66 ± 0.03	0.03	400.43 ± 0.02	408.00 ± 0.01
XB2	3.92 ± 0.02	0.11	384.06 ± 0.05	381.00 ± 0.02
XB3	4.39 ± 0.01	0.06	403.95 ± 0.01	398.16 ± 0.03
YB1	4.61 ± 0.04	0.04	438.42 ± 0.04	437.75 ± 0.05
YB2	4.90 ± 0.01	0.01	422.04 ± 0.02	445.45 ± 0.03
YB3	4.57 ± 0.02	0.02	374.50 ± 0.04	383.36 ± 0.03
YH1	4.62 ± 0.01	0.02	331.78 ± 0.05	337.78 ± 0.02
YH2	4.67 ± 0.01	0.02	295.72 ± 0.02	298.56 ± 0.04
YH3	3.29 ± 0.01	0.10	230.08 ± 0.01	226.20 ± 0.01
ZR1	4.34 ± 0.03	0.04	343.02 ± 0.03	365.72 ± 0.02
ZR2	4.76 ± 0.01	0.02	375.14 ± 0.02	379.13 ± 0.01
ZR3	4.68 ± 0.01	0.02	371.94 ± 0.05	369.56 ± 0.03

Notes: DD stands for “Dongde Lake”, KM stands for “Kongma Township”, LR stands for “Longren Township”, SP stands for “Sapu Glacier”, WQ stands for “Wuqiong Lake”, NM stands for “Nima County”, PR stands for “Puruogangri Glacier”, SH stands for “Shuanghu County”, XB stands for “Xiongba Township”, YB stands for “Yangbajing Town”, YH stands for “Yanhu Township”, and ZR stands for “Zaren Township”.

## Data Availability

The results of this study have been submitted to the GenBank database under accession number PRJNA700796.
